# Factors associated with hospital and intensive care admission in paediatric SARS-CoV-2 infection: a prospective nationwide observational cohort study

**DOI:** 10.1007/s00431-021-04276-9

**Published:** 2021-11-29

**Authors:** Anita Uka, Michael Buettcher, Sara Bernhard-Stirnemann, Yves Fougère, Dehlia Moussaoui, Lisa Kottanattu, Noémie Wagner, Petra Zimmermann, Nicole Ritz, Swiss Paediatric Surveillance Unit (SPSU), M. Albisetti, V. Bernet, C. Betti, F. Cachat, P. Caplazi, M-L. Decker, E. Durrer, S. Fluri, M. Gebauer, M. Gehri, E. Giannoni, S. Grupe, M. Horn, A. L’Huiller, T. Karen, E. Kellner, G. Laube, B. Laubscher, J. Llor, F. Luterbacher, H. Madlon, A. Malzacher, M. Martins, J. McDougall, A. Merglen, S. Minocchieri, V. Muehlethaler, T. Neuhaus, A. Niederer, S. Nikorelou, M. Plebani, Ratnasabapathy, C. Relly, T. Riedel, M. Russo, H. Schmid, K. Staudacher, M. Torres Escobar, J. Wildhaber, A. Wörner, A. Zemmouri

**Affiliations:** 1 Faculty of Science and Medicine, University of Fribourg, Fribourg, Switzerland; 2 Department of Paediatrics, Fribourg Hospital HFR, Fribourg, Switzerland; 3 Paediatric Infectious Diseases, Children’s Hospital Lucerne, Luzern, Switzerland; 4 Department of Paediatrics, Children’s Hospital, Cantonal Hospital Aarau, Aarau, Switzerland; 5 Pediatric Infectious Diseases and Vaccinology Unit, Department Women-Mother-Child, Lausanne University Hospital, Lausanne, Switzerland; 6 Department of Paediatrics, Gynaecology and Obstetrics, General Paediatrics Division, Geneva University Hospitals, Geneva, Switzerland; 7 Ente Ospedaliero Cantonale, Istituto Pediatrico Della Svizzera Italiana, Bellinzona, Switzerland; 8 Infectious Diseases Research Group, Murdoch Children’s Research Institute, Parkville, Australia; 9 Paediatric Infectious Diseases and Vaccinology, University of Basel Children’s Hospital, Basel, Switzerland; 10 Department of Paediatrics, The Royal Children’s Hospital Melbourne, The University of Melbourne, Melbourne, Australia; 11 University Children’s Hospital Basel UKBB, Spitalstrasse 33, CH-4031 Basel, Switzerland

**Keywords:** COVID-19, Child, Epidemiology, Clinical presentation, Outcome, Transmission

## Abstract

Coronavirus disease 2019 (COVID-19) is usually less severe in children compared to adults. This study describes detailed clinical characteristics, treatment and outcomes of children with COVID-19 in a non-hospitalised and hospitalised setting and quantifies factors associated with admission to hospital and intensive care unit in children with SARS-CoV-2 infection on a nationwide level. Data were collected through the Swiss Paediatric Surveillance Unit from children < 18 years with confirmed SARS-CoV-2 infection. All 33 paediatric hospitals in Switzerland reported non-hospitalised and hospitalised cases from March 1 to October 31, 2020 during both pandemic peaks. In total, 678 children were included. The median age was 12.2 years (IQR 5.0–14.6), 316 (46.6%) were female and 106 (15.6%) had comorbidities. Overall, 126 (18.6%) children were hospitalised of whom 16 (12.7%) required ICU admission. Comorbidities were the only factor associated with hospital admission in a multivariable regression analysis (odds ratio 3.23, 95%CI 1.89 to 5.50; *p*-value < 0.01). Children with preexisting comorbidities did not require ICU admission more often. Hospitalised children more often presented with fever (96 [76.2%] vs 209 [38.1%], *p*-value < 0.01) and rash (16 [12.8%] vs 6 [1.1%], *p*-value < 0.01). Anosmia/dysgeusia was more prevalent in non-hospitalised children (73 [13.3%] vs 3 [2.4%], *p*-value < 0.01). In hospitalised children, oxygen treatment was required in 34 (27.0%), inotropes in nine (7.3%) and mechanical ventilation in eight (6.3%) cases. Complications were reported in 28 (4.1%) children with cardiovascular complications being most frequent (12 [1.8%]). Three deaths were recorded.

*Conclusion*: This study confirms that COVID-19 is mostly a mild disease in children. Fever, rash and comorbidities are associated with higher admission rates. Continuous observation is necessary to further understand paediatric COVID-19, guide therapy and evaluate the necessity for vaccination in children.

**Table:** 

**What is Known:** *• Clinical manifestations of SARS-CoV-2 infection in children vary from asymptomatic to critical disease requiring intensive care unit admission.* *• Most studies are based on hospitalised children only; currently, there is limited data on non-hospitalised children.*	
**What is New:** *• The clinical spectrum and severity of COVID-19 is influenced by age: in children less than 2 years, fever, cough and rhinorrhoea are the most common symptoms and in adolescents, fever, cough and headache are more common.* *• Hospitalised children more often presented with fever and rash, while anosmia/dysgeusia is more prevalent in non-hospitalised children.* *• Children with pre-existing comorbidities are more frequently hospitalised but do not require ICU admission more often.*

**Supplementary Information:**

The online version contains supplementary material available at 10.1007/s00431-021-04276-9.

## Introduction

Compared to adults, coronavirus disease 2019 (COVID-19) manifests differently in infants, children and adolescents [1–5]. Although, the disease severity is often milder in children, paediatric patients may also develop severe disease requiring admission to intensive care unit (ICU) and may very rarely die from COVID-19 [6–8]. Additionally, children presenting with a delayed inflammatory disease called ‘paediatric inflammatory multisystem syndrome-temporally associated with SARS-CoV-2 (PIMS-TS)’ or’multisystem inflammatory syndrome in children (MIS-C)’ have been reported [7, 9–15].

To date, the data on SARS-CoV-2 infection in children and adolescents either come from non-hospitalised children with limited clinical information or from hospitalised children only. This limits the knowledge in paediatric COVID-19 on factors associated with admission including clinical presentation and risk factors such as age, sex or comorbidities. This study presents epidemiological data from active surveillance of SARS-CoV-2 infections in non-hospitalised and hospitalised children in Switzerland and provides insights regarding risk factors for admission.

## Methods

### Study design and population

Our study is a prospective nationwide observational cohort study that describes detailed clinical characteristics and outcomes of children with laboratory-confirmed COVID-19 in a non-hospitalised and hospitalised setting. Paediatric SARS-CoV-2 infections are actively monitored in an observational study by the Swiss Paediatric Surveillance Unit (SPSU, http://www.spsu.ch) since March 2020. The current analysis includes data from March 1 to October 31, 2020. All 33 paediatric and neonatological hospitals in Switzerland participated and notify cases monthly. Upon notification, the investigators sent the reporting centres an electronic clinical report form through RedCap or in paper form (see supplementary data Questionnaire) [16]. All data were recorded anonymously and reviewed by the investigators and further clarified with the reporting physician when needed. The study has received ethical approval by the Ethikkommission Nordwest- und Zentralschweiz (EKNZ 2020–01,130).

### Case definition

Children and adolescents < 18 years of age who presented to a Swiss paediatric hospital and received ambulatory or hospitalised care were included if diagnosed with COVID-19 by detection of SARS-CoV-2 from a clinical specimen using a validated polymerase chain reaction (PCR) test or serology. Retrospective screening of patients with the following criteria was used for identification of potential PIMS-TS cases: PIMS-TS reported by the clinician, SARS-CoV-2 serology performed, ICU admission or cardiac changes.

### Statistical analysis

Continuous data were summarised using median and interquartile ranges. Categorical data were presented as percentage. Categorical data were compared using the *χ*^2^ test, with *p*-values < 0.05 considered as significant. Co-occurrence symptoms were clustered using the K-means clustering solution and visualised with a heat map. A multivariable logistic regression model of risk of admission was fitted by including all variables used for univariable analysis origin. For the assessment of age, the following groups were made 0 to < 2, 2 to < 5, 5 to < 10 and 10 to < 18 years of age. R (Version 1.2.5019) was used for statistical analyses.

## Results

### Study population

**Fig. 1:**
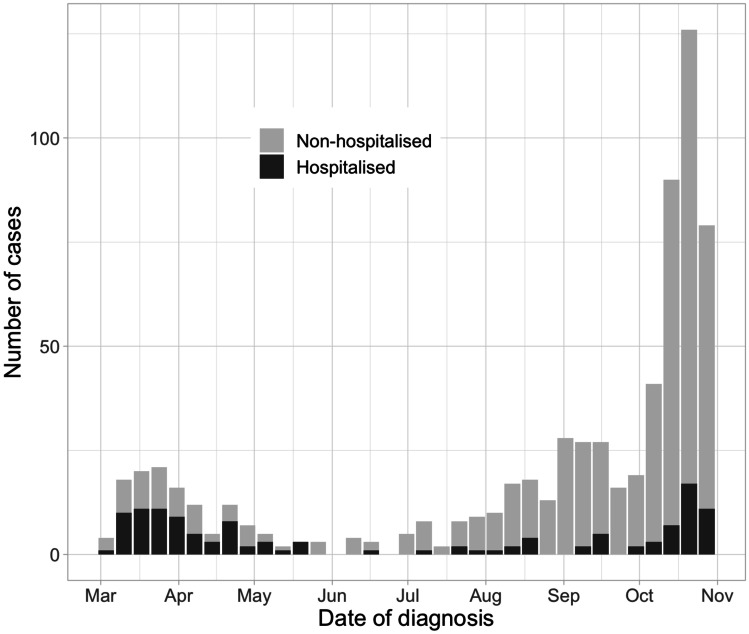
Number of children with SARS-CoV-2 infection over time

A detailed dataset was returned for 682 cases, of which 678 were included in the final analysis. Reasons for exclusion were duplication in reporting (*n* = 3) and age ≥ 18 years (*n* = 1). The age of the children ranged from 7 days to 17.9 years with a median of 12.2 years (interquartile range (IQR) 5.0–14.6) (Table 1). Most of the children were Caucasian (532 [78.5%]), followed by Arabic (29 [4.3%]), Hispanic (27 [4.0%]), Black (18 [2.7%]) and Asian (10 [1.5%]). Ethnicity was unknown for 62 (9.1%) children. Numbers of reported children over time are shown in Fig. 1 and stratified to age group in supplementary data Fig. S1. Geographical and temporal distribution of SARS-CoV-2 cases in Swiss cantons (political states) is shown in supplementary data Fig. S2.

**Table 1:** Baseline characteristics and clinical information of children with SARS-CoV-2 infection

	**Overall**	**Non-hospitalised** ***n* (%)**	All hospitalised* n (%)	**ICU**
	***n* (%)**			***n* (%)**
	***n* = 678**	***n* = 552**	***n* = 126**	***n* = 16**
**Age in years**				
< 2	117 (17.3)	52 (9.4)	65 (51.6)	4 (25.0)
2 to < 5	49 (7.2)	42 (7.6)	7 (5.6)	0 (0.0)
5 to < 10	99 (14.6)	85 (15.4)	14 (11.1)	2 (12.5)
≥ 10	413 (60.9)	373 (67.6)	40 (31.7)	10 (62.5)
**Age < 1 month**	17 (2.5)	5 (0.9)	12 (9.5)	1 (6.2)
**Female**	316 (46.6)	262 (47.5)	54 (42.9)	5 (31.3)
**Comorbidities**	106 (15.6)	72 (13.0)	34 (27.0)	5 (31.3)
**Symptoms**				
Fever	305 (45.3)	209 (38.1)	96 (76.2)	11 (68.8)
Cough	277 (41.2)	229 (41.8)	48 (38.4)	4 (26.7)
Rhinorrhoea	191 (28.4)	142 (25.9)	49 (39.2)	4 (26.7)
Pharyngitis	187 (27.8)	164 (29.9)	23 (18.4)	3 (20.0)
Anosmia/dysgeusia	76 (11.3)	73 (13.3)	3 (2.4)	1 (6.7)
Abdominal pain	76 (11.3)	60 (10.9)	16 (12.8)	5 (33.3)
Diarrhoea	68 (10.1)	46 (8.4)	22 (17.6)	5 (33.3)
Vomiting	59 (8.8)	37 (6.8)	22 (17.6)	5 (33.3)
Respiratory distress	49 (7.3)	18 (3.3)	31 (24.6)	10 (62.5)
Rash	22 (3.3)	6 (1.1)	16 (12.8)	5 (33.3)
Oxygen saturation < 92%	18 (2.7)	1 (0.2)	17 (13.5)	6 (37.5)
Asymptomatic	39 (5.8)	35 (6.3)	4 (3.2)	0 (0.0)

*ICU* intensive care unit

^*^Numbers of hospitalised children include those admitted to ICU

### Hospitalisation and management

Overall, 126 (18.6%) children were hospitalised of which 14 (11.1%) were hospitalised for other reasons than infection with SARS-CoV-2. A total of 16 (12.7%) children required ICU admission for the following reasons: hemodynamic instability (*n* = 8), respiratory failure (*n* = 4), prematurity (*n* = 1), coma (*n* = 1), cardiovascular arrest (*n* = 1), neurogenic shock (*n* = 1). One adolescent with a mild upper respiratory tract illness was admitted to ICU for a non-COVID-19-related reason (neurogenic shock after an accident). Ethnicity of the children admitted to ICU were Caucasian (*n* = 8), Black (*n* = 4), Hispanic (*n* = 3) and unknown (*n* = 1). Oxygen was required in 34 (27.0%), inotropes in nine (7.3%) and mechanical ventilation in eight (6.3%) hospitalised cases. Complications were reported in 25 (19.8%) hospitalised children with cardiovascular complications being most frequent (11 [8.7%]). A total of 48 children were retrospectively analysed for potential PIMS-TS of which 17 children were identified as cases based on available data (eight non-ICU admitted, nine ICU admitted). Three deaths were recorded.

Overall, most children (646 [95.3%]) did not receive medication. Specific treatment was given to 10 (1.8%) of non-hospitalised, 15 (13.6%) of hospitalised and 12 (75.0%) of ICU-admitted children. Among hospitalised children (non-ICU admitted), six (5.5%) received corticosteroids, two (1.8%) each hydroxychloroquine and intravenous immunoglobulins (IVIG) and one (0.9%) tocilizumab. Among ICU-admitted children, nine (56.3%) received biologicals (anakinra (*n* = 7), tocilizumab (*n* = 2)), seven (43.8%) each corticosteroids, IVIG and two (12.5%) hydroxychloroquine. A combination of IVIG and corticosteroids was given to five children with suspected PIMS-TS in the ICU group. No further treatment including remdesivir was given (supplementary data Questionnaire). The median duration of hospitalisation for non-ICU admitted children was 3.0 days (IQR 2.0–4.0) and for children admitted to ICU 14 (IQR 4.75–15.25) days (supplementary data Fig. S3).

### Comorbidities

A total of 106 (15.6%) children had pre-existing medical conditions, the most common comorbidities beeing: respiratory (45 [42.5%]), endocrinology (15 [14.2%]), haemato-oncology (12 [11.3%]) and cardiovascular (10 [9.4%]) (details are listed in Table 2). Hospitalised children had significantly more comorbidities than non-hospitalised children (*p*-value < 0.01). Five (31.3%) children admitted to ICU had pre-existing comorbidities: three children had asthma/bronchitis, a neonate had apnoea of prematurity (born at 29 weeks gestational age) and a 2-month-old infant had isolated microcephaly with a normal cerebral ultrasound. Children requiring admission to ICU did not have pre-existing medical conditions more frequently compared to non-ICU-hospitalised children. A univariable regression analysis showed that children ≥ 2 years of age were less likely to be hospitalised, and comorbidities increased the risk of hospitalisation in both uni- and multivariable regression analysis (Table 3).

**Table 2:** Comorbidities in children with SARS-CoV-2 infection. Note that some patients had more than one comorbidity reported

	**Overall**	**Non-hospitalised**	Hospitalised*	**ICU**
	***n (%)***	***n*** (%)	***n*** (%)	***n*** (%)
**Any pre-existing comorbidities**	**106 (100)**	**72 (13)**	**34 (27.0)**	**5 (31.3)**
**Respiratory disease**	**45 (42.5)**	**31 (43.1)**	**14 (41.2)**	**4 (80.0)**
Asthma/bronchitis	38	30	8	3
Obstructive sleep apnoea/adenoid hypertrophy	2	0	2	0
Cystic fibrosis/primary ciliary dyskinesia	2	0	2	0
Apnoea of prematurity	2	0	2	1
Bronchopulmonary dysplasia	1	1	0	0
**Endocrinological disease**	**15 (14.2)**	**12 (16.7)**	**4 (11.8)**	**0**
Obesity	10	8	2	0
Diabetes mellitus type 1	5	3	2	0
Hashimoto thyroiditis	1	1	0	0
**Haemato-oncological disease**	**12 (11.3)**	**5 (6.9)**	**7 (20.6)**	**1 (20.0)**
Leukaemia	5	2	3	0
Thalassemia/sickle-cell disease	2	1	1	0
Neutropenia	2	0	2	1
Medulloblastoma	1	1	0	0
Severe anaemia	1	0	1	0
Glucose-6-dehydrogenase deficiency	1	1	0	0
**Cardiovascular disease**	**10 (9.4)**	**5 (6.9)**	**5 (14.7)**	**0**
Congenital heart defect	6	2	4	0
Post heart transplant	1	0	1	0
Myocarditis	1	1	0	0
Hypertrophic cardiomyopathy	1	1	0	0
Familial long QT syndrome	1	1	0	0
**Neurologic disease**	**8 (7.5)**	**4 (5.6)**	**4 (11.8)**	**1 (20.0)**
Psychiatric	2	0	2	0
Epilepsy	2	2	0	0
Microcephaly	1	0	1	1
Neurofibromatosis	1	1	0	0
Multiple strokes	1	0	1	0
Autistic spectrum disorder	1	1	0	0
**Surgical comorbidities**	**6 (5.7)**	**5 (6.9)**	**1 (2.9)**	**0**
**Immunodeficiency**	**5 (4.7)**	**2 (2.8)**	**3 (8.8)**	**0**
Commune variable immune deficiency	1	0	1	0
Microdeletion 22q11	1	0	1	0
Autoimmune lymphoproliferative syndrome	1	1	0	0
Complement activation deficiency	1	1	0	0
Status post renal transplant	1	0	1	0
**Nephrological disease**	**5 (4.7)**	**1 (1.4)**	**4 (11.8)**	**0**
Urolithiasis	1	0	1	0
Hydronephrosis	1	1	0	0
Kidney failure	2	0	2	0
Autosomal recessive polycystic kidney disease	1	0	1	0
**Genetic disease**	**4 (3.8)**	**2 (2.8)**	**2 (5.9)**	**0**
Von Hippel Lindau	1	1	0	0
Down disease	2	1	1	0
Mowat Wilson syndrome	1	0	1	0
**Auto-immune disease**	**4 (3.8)**	**3 (4.2)**	**1 (2.9)**	**0**
Juvenile idiopathic arthritis	3	3	0	0
Granulomatosis with polyangiitis	1	0	1	0
**Auto-inflammatory disease**	**3 (2.8)**	**3 (4.2)**	**0**	**0**
Cryopyrin-associated periodic syndrome	1	1	0	0
Familial Mediterranean fever	1	1	0	0
Chronic multifocal osteomyelitis	1	1	0	0
**Gastroenterological disease**	**2 (1.9)**	**2 (2.8)**	**0**	**0**
Gastritis	1	1	0	0
Coeliac disease	1	1	0	0
**Hepatological disease**	**2 (1.9)**	**2 (2.8)**	**0**	**0**
Cholestasis	2	2	0	0
**Prematurity**	**2 (1.9)**	**1 (1.4)**	**1 (2.9)**	**1**

^*^Numbers of hospitalised children include those admitted to intensive care unit (ICU)

**Table 3:** Univariable and multivariable regression analysis for risk of hospitalisation. Note, the number of specific comorbidities was too low to be included in the multivariable analysis

	**Univariable regression**			**Multivariable regression**		
	**OR**	**95% CI**	***p*-value**	**OR**	**95% CI**	**p-value**
Age < 2 years (Reference)	1.23	0.85 to 1.78	0.27	1.52	0.72 to 3.24	0.27
Age 2 to < 5 years	0.14	0.05 to 0.31	** < 0.01**	0.10	0.04 to 0.24	** < 0.01**
Age 5 to < 10 years	0.13	0.07 to 0.26	** < 0.01**	0.11	0.05 to 0.22	** < 0.01**
Age > 10 years	0.09	0.05 to 0.14	** < 0.01**	0.08	0.05 to 0.13	** < 0.01**
Gender (male)	0.81	0.55 to 1.20	0.31	0.78	0.50 to 1.21	0.27
Comorbidities (Any)	2.49	1.55 to 3.95	** < 0.01**	3.23	1.89 to 5.50	** < 0.01**
Cardiac comorbidities	4.56	1.25 to 16.63	0.02	–	–	–
Respiratory comorbidities	2.12	1.06 to 4.04	0.03	–	–	–
Immunodeficiency	6.76	1.11 to 51.75	0.04	–	–	–
Haemato-oncological comorbidities	6.49	2.04 to 22.26	** < 0.01**	–	–	–

### Symptoms

**Fig. 2:**
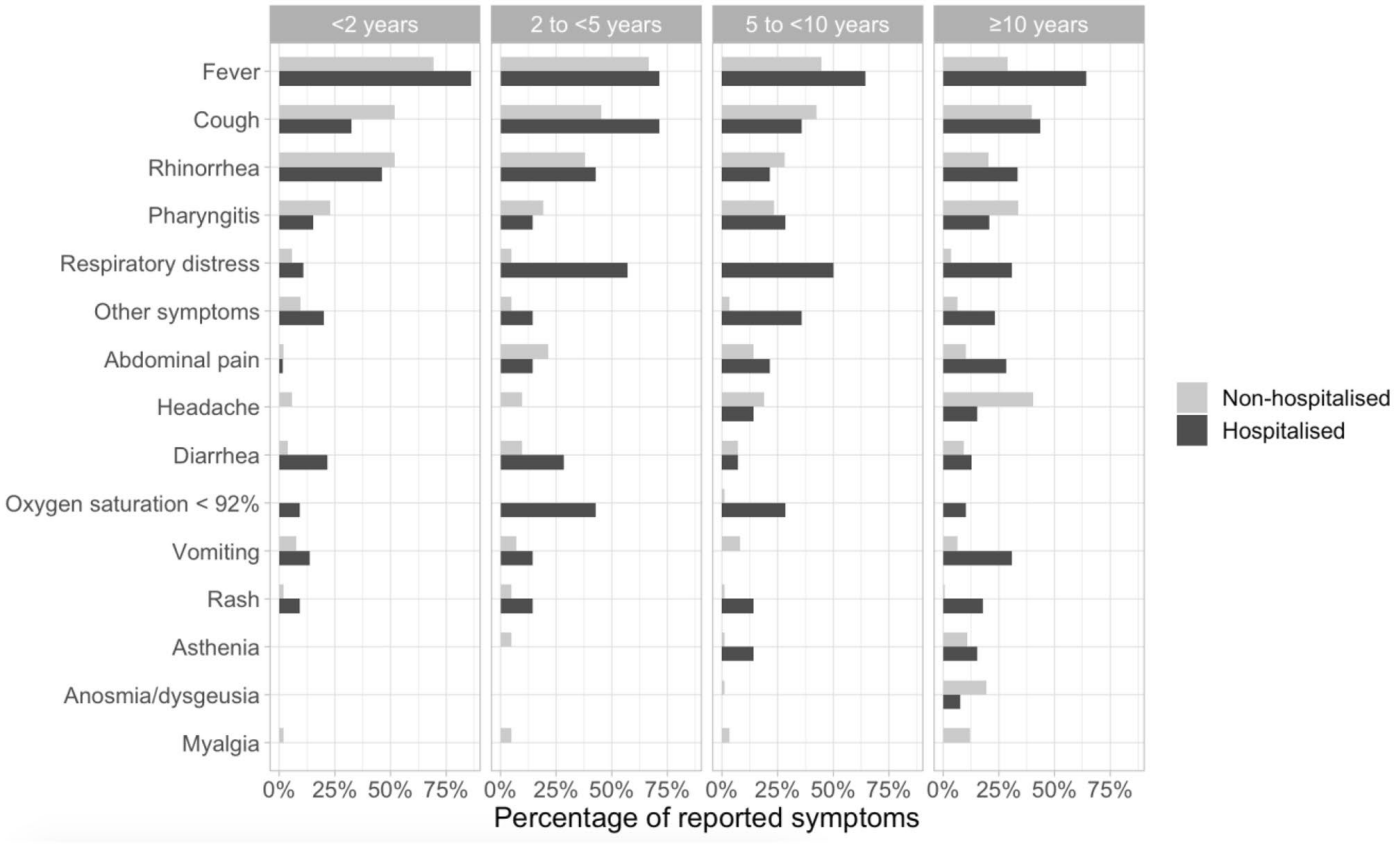
Symptoms distribution in non-hospitalised and hospitalised children with SARS-CoV-2 infections according to age group. Other symptoms included conjunctivitis, otalgia, cheilitis, hand oedema, thoracic pain, arthralgia, acrocyanosis, fainting, seizure, alguria, orchitis and macro-haematuria

**Fig. 3:**
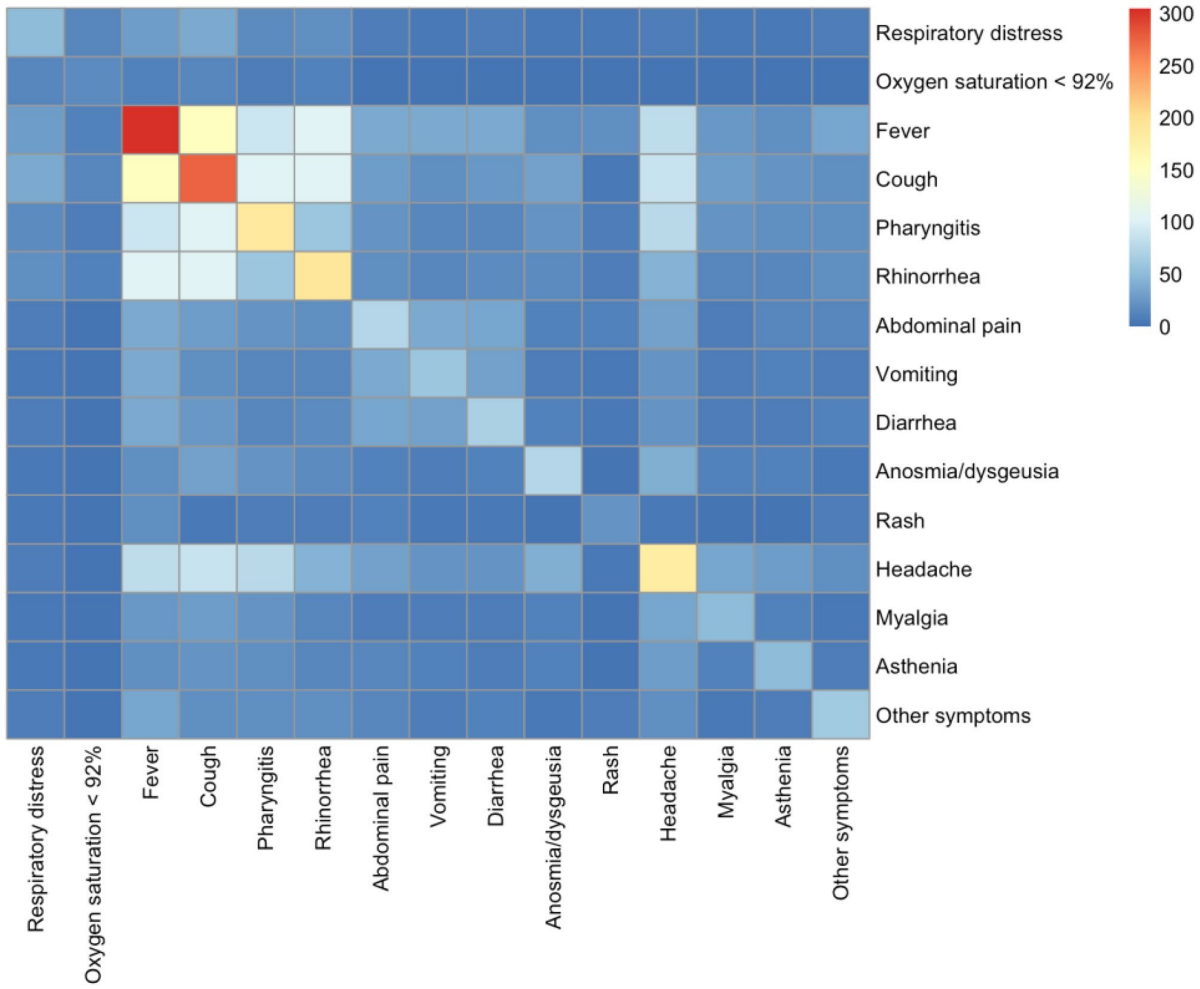
Heatmap using k-means with symptom cluster (the colour code stands for the number of children with corresponding symptoms)

Overall, fever was the most frequent symptom observed among children with COVID-19 (305 [45.3%]) (Table 1). In children aged less than 2 years of age, fever, cough and rhinorrhoea were the most common symptoms, and in adolescents between 10 and 18 years of age, fever, cough and headache were more commonly reported (a detailed distribution of symptoms according to age group is presented in Fig. 2). Fever and rash were more common in hospitalised compared with non-hospitalised children ((96 [76.2%] vs 209 [38.1%], *p*-value < 0.001) and (16 [12.8%] vs 6 [1.1%], *p*-value < 0.001), respectively. In contrast, anosmia/dysgeusia was more prevalent in non-hospitalised children (73 [13.3%] vs 3 [2.4%], *p*-value 0.001). Children admitted to ICU more often had abdominal pain (5 [33.3%] vs 11 [10.0%], *p*-value 0.034) and rash (5 [33.3%] vs 11 [10.0%], *p*-value 0.034) than non-ICU-hospitalised children. A heatmap with a co-occurrence matrix for symptoms showed three clusters of symptoms representing three different clinical phenotypes (Fig. 3). The first cluster represents an upper respiratory tract illness with fever, cough, rhinorrhoea and pharyngitis; the second a gastrointestinal illness with abdominal pain, diarrhoea and vomiting, and the third cluster corresponds to more constitutional symptoms with headache, myalgia and asthenia.

### Complications

A total of 28 (4.1%) children with SARS-CoV-2 infection developed complications; this was more frequent in hospitalised than non-hospitalised children (*p*-value < 0.001). The most frequent complications/non-pulmonary organ manifestations were cardiovascular in 12 (1.8%) children, including coronary artery dilatation (*n* = 4), elevation of cardiac enzymes (*n* = 3), hypotensive shock (*n* = 3), myocarditis (*n* = 1), vasculitis (*n* = 3) and thromboembolism (*n* = 1). Bacterial co-infection was reported in 8 (1.2%) children, exclusively in hospitalised children. Further complications were pancytopenia (*n* = 5), kidney failure (*n* = 4), seizures (*n* = 3), encephalopathy (*n* = 1), polyradiculoneuritis (*n* = 1) and myopathy (*n* = 1). No nosocomial infections were reported.

In hospitalised children, three (2.4%) deaths were reported during the study period. A 10-month-old infant with severe brain oedema, signs of hypoxic-ischemic encephalopathy and cerebral haemorrhages presenting with vomiting without fever, respiratory distress (with evidence of respiratory illness on chest radiography) and status epilepticus. A 2-month-old infant with cardiorespiratory arrest secondary to cerebral haemorrhage presenting with acholic stools, haematochezia, haematemesis and hepatosplenomegaly with abnormal liver function. A 6-year-old with an Epstein-Barr Virus (EBV)-associated hemophagocytic lymphohistiocytosis developed cerebral haemorrhage due to coagulation deficiency caused by liver failure. He presented with abdominal pain, fever and abnormal liver function 2 weeks after a SARS-CoV-2 infection and was initially suspected to have PIMS-TS.

### Diagnosis

Diagnosis of SARS-CoV-2 infection was mostly confirmed by PCR (663 [98.1%]). Of the 40 children in whom serology was done, 35 (87.5%) were positive. Five ICU-admitted patients had positive serology with negative PCR. A chest radiography was done in 47 (6.9%) children and showed unilateral and bilateral changes in six (12.8%) and 16 (34.0%) cases, respectively. Echocardiography was done in 47 (6.9%) children; abnormal findings were identified in eight (21.6%) children (coronary dilatation (*n* = 4), reduced left ventricle ejection fraction (*n* = 3) and dyskinesia (*n* = 1)), and all of them were hospitalised. Other diagnostic tests included abdominal ultrasound (16 [2.4%]) and thoracic computed tomography (9 [1.3%]) (further details in supplementary data Table S1).

### Transmission

In total, 309 children (45.6%) had a family member with a confirmed or suspected SARS-CoV-2 infection (supplementary data Fig. S4). Community-acquired infection (including school and day-care) was confirmed or suspected in 86 (12.7%) children. In one-third of children, the primary case was unknown 284 (41.9%).

## Discussion

This nationwide study presents the first epidemiological data from active surveillance of SARS-CoV-2 infection in non-hospitalized and hospitalized children in Switzerland. Data have been collected during both pandemic waves: the first wave was marked by general lockdown with school closures. The second wave was characeristed by an important rise of cases in October, which was similar to the evolution of the pandemic in the adult population and was followed by a second lockdown in November [17]. Our findings suggest that comorbidities are an important factor associated with two times higher hospitalisation rates in children and adolescents. Data from the Centres of Disease Control surveillance of in the USA suggest that overall hospitalisation rates are six times higher among children with a reported underlying condition [18]. In children ≤ 9 years of age, the rate of underlying conditions was reported to be 4.1% overall and 22.3% in those hospitalised. We have found similar rates of comorbidities in hospitalised but higher rates in non-hospitalised children. The latter may be explained by the fact that our data are based on a hospital-centred surveillance, which likely underestimates the burden of disease in non-hospitalised children. In addition, children with comorbidities were more likely tested and referred to hospitals, particularly in the early phase of the pandemic. This is also reflected by the fact that we observed a more severe spectrum of paediatric COVID-19 with a lower rate of asymptomatic children compared to other studies [19, 20]. Another paediatric study from the UK reported much higher rates of comorbidities of 42% in hospitalised children, but this study did not include data on non-hospitalised cases [1]. The most frequent comorbidities reported in the UK study were neurological, asthma and haemato-oncological or immunological which compares to the spectrum reported in our study. Whether underlying conditions are associated with increased severity of disease or a lower threshold for admission remains unclear based on our data.

 In our setting, 13% of hospitalised children required admission to ICU, which is in line with other European studies [1, 19, 21]. Similarly, to other studies, children with serious medical conditions (immunodeficiency, haemato-oncological, cardiac or metabolic disease) in our cohort did not develop severe COVID-19 requiring ICU admission more often than previously healthy children [22]. A recent systematic review suggests that children with underlying cardiac disease might be at increased risk of severe COVID-19 [23]. However, we did not find this in our study population, where children with underlying cardiac disease (including one patient after heart transplantation) did not experience a severe disease course. Three children with asthma/bronchitis required ICU admission due to respiratory failure. Evidence are sparse on whether asthma is a risk factor for severe COVID-19 in children; a systematic review found only two reports considering asthma as a risk factor for SARS-CoV-2 infection [24]. However, a large US retrospective paediatric study found that the risk of testing positive for SARS-CoV-2 was reduced in children with a respiratory disorder, including asthma [25]. Moreover, children with asthma are underrepresented during this pandemic, and studies in adults have not identified SARS-CoV-2 as a trigger for asthma exacerbations [26]. Several hypotheses have been proposed to explain this unexpected finding, including an impaired immune response to SARS-CoV-2. Children with asthma have lower levels of interferon-gamma and consequently reduced angiotensin-converting enzyme 2 (ACE-2) gene expression in airway epithelium, which act as the entry point of SARS-CoV-2 into the human body [26]. Other studies report reduced ACE-2 expression in patients with allergic asthma or after inhaled corticosteroids [27, 28].

Our study clearly shows that the clinical spectrum of COVID-19 is different in hospitalised and non-hospitalised children with fever and rash being more common in those admitted, but cough, rhinorrhoea and pharyngitis being comparable. Although fever was the most frequent symptom, especially among young children, the incidence was lower than reported by other multicentric studies [1, 19, 21]. This lower prevalence might reflect the higher proportion of adolescents in our cohort. Age is a further important factor influencing the clinical spectrum of disease with fever and respiratory symptoms of lower severity being more frequent in children below 2 years of age. Older children more likely present with non-respiratory symptoms, and anosmia/dysgeusia was only rarely recorded in children less than 10 years of age. A similar spectrum was described in the UK paediatric cohort; however, this only included hospitalised children [1].

Our findings confirm that paediatric COVID-19 is mostly a mild illness. Several hypothesis have been proposed to explain the milder disease seen in children, including an age-related difference in the immune response, with a stronger innate immune response in children and age-related differences in expression of ACE-2 [5, 29]. Our data also captures cases with the suspicion of PIMS-TS corresponding to the most severe spectrum of COVID-19. Of the three deaths reported, two were being temporally related to SARS-CoV-2 infection without another disease identified.

Evidence-based treatment options for paediatric COVID-19 are still lacking [30]. In our study, the majority of non-hospitalised but also hospitalised children did not receive specific treatment. However, children admitted to ICU with PIMS-TS required inotropic support and received systemic corticosteroid and IVIG in combination with immunomodulators (including anakinra (interleukin-1 inhibitor) and tocilizumab (interleukin-6 inhibitor)). Similarly, most case series report using IVIG and/or corticosteroid as main therapeutic option for children with PIMS-TS features, given the similitude with Kawasaki disease [14, 15, 31–34]. The rational for using anti-cytokine agents (anakinra or tocilizumab) is that these toxic shock-like clinical presentations are caused by exaggerated immune response and excessive cytokine release, called cytokine storm, rather than by viral replication itself [35]. However, there are insufficient data to recommend for or against the use of specific treatment for PIMS-TS, and management remains centred on effective supportive care [30].

The strengths of our study are the multicentre nationwide study design capturing the widespread epidemiology of SARS-CoV-2 infection in a European country, the inclusion of a large number of children including non-hospitalised children and the detailed demographic and clinical data. However, our study overestimates the admission rate as we did not include patients tested in private practices and as some children were hospitalised for reasons other than SARS-CoV-2 infection. Furthermore, the threshold for admission might have been different depending on local guidelines, pre-existing comorbidities, physicians’ discretion and presentations outside office hours. Data from the Federal Office of Public Health show a 0.9% admission rate in children in the same time period (personal communication from Mirjam Mäusezahl, Federal Office of Public Health, 23 March 2021). In addition, in the early weeks of the COVID-19 pandemic, SARS-CoV-2 testing was not considered a priority in children, and only children with severe symptoms or persistent fever were tested. Testing strategies also differed regionally which may have affected some of the observed regional differences. Analysis of weight and obesity as a potential risk factor for hospitalisation could not be included as weight was not recorded in most non-hospitalised cases. We are also unable to analyse children classified as PIMS-TS in detail as specific data on PIMS-TS cases are only prospectively collected since November 2020. Due to the important rise of cases in October 2020, data collection of SPSU SARS-CoV-2 for non-hospitalised children was stopped, and therefore, we are unable to include non-hospitalised children in the data analysis during the further second wave.

## Conclusion

This study confirms that COVID-19 is mostly a mild disease in children and adolescents with low mortality. Fever, rash and comorbidities are associated with higher admission rates. The clinical spectrum and severity are influenced by age in paediatric COVID-19. Continuous observation is necessary to further understand paediatric COVID-19, guide therapy and evaluate the necessity for vaccination in children.

## Supplementary information

Below is the link to the electronic supplementary material.

Supplementary file1 (PDF 102 KB)

## Data Availability

Data collected for the study and the study protocol will be made available to others on request.

## Abbreviations

ACE-2: Angiotensin-converting enzyme 2
COVID-19: Coronavirus disease-2019
EBV: Epstein-Barr Virus
ICU: Intensive care unit
IQR: Interquartile range
IVIG: Intravenous immunoglobulins
MIS-C: Multisystem inflammatory syndrome in children
PIMS-TS: Paediatric inflammatory multisystem syndrome-temporally associated with SARS-CoV-2
PCR: Polymerase chain reaction
SARS-CoV-2: Severe acute respiratory syndrome coronavirus 2
SPSU: Swiss Paediatric Surveillance Unit
