# Supplementary material for: Factors associated with hospital and intensive care admission in paediatric SARS-CoV-2 infection: a prospective nationwide observational cohort study
Source: Eur J Pediatr. 2021 Nov 29;181(3):1245–55. doi: 10.1007/s00431-021-04276-9 (PMC8628837; doi:10.1007/s00431-021-04276-9)
Supplement: Supplementary file 1 — Supplementary file1 (PDF 102 KB) [file 431_2021_4276_MOESM1_ESM.pdf]

SARS-CoV-2 Infection Questionnaire

Thank you very much for completing the questionnaire

If you need to interupt filling it in and want to come back later (e.g. because of missing information) you can do so by emailing yourself a link at the bottom of the questionnaire

In case of questions please contact SPSU-corona@ukbb.ch

PD Dr. med. N. Ritz, Universitäts-Kinderspital beider Basel (UKBB)

Dr. phil. Dr. med. P. Zimmermann, Kantonsspital Fribourg (HFR)

Baseline data

Reporting hospital	◯ Aarau (Code SPSU: 3123)<br>◯ Baden (Code SPSU 1139)<br>◯ Basel (Code SPSU 1121)<br>◯ Bern (Code SPSU 3117)<br>◯ Biel (Code SPSU 1114)<br>◯ Centre hospitalier Rennaz (Aigle, Meryin, Vevey) (Code SPSU 2108)<br>◯ Chur (Code SPSU 1129)<br>◯ Delémont (Code SPSU 2115)<br>◯ Fribourg (Code SPSU 2107)<br>◯ Genève (Code SPSU 2104)<br>◯ CHUV, Lausanne (Code SPSU 2202)<br>◯ Hôpital de l'Enfance, Lausanne (Code SPSU 2101)<br>◯ Istituto Pediatrico della Svizzera Italiana (Bellinzona, Locarno, Lugano) (Code SPSU 3125)<br>◯ Luzern (Code SPSU 1124)<br>◯ Morges (Code SPSU 2103)<br>◯ Münsterlingen (Code SPSU 1136)<br>◯ Neonatologie, Universitäts-Kinderklinik, Bern (Code SPSU 3118)<br>◯ Neonatologie, Frauenklinik, Kantonsspital, St. Gallen (Code SPSU 1138)<br>◯ Neonatologie, Dép. Femme-Mère-Enfant, CHUV (Code SPSU 2102)<br>◯ Neonatologie, Universitäts-Frauenklinik, Zürich (Code SPSU 3134)<br>◯ Neuchâtel (Code SPSU 2112)<br>◯ St. Gallen (Code SPSU 3117)<br>◯ Sion (Code SPSU 2111)<br>◯ Visp (Code SPSU 1119)<br>◯ Winterthur (Code SPSU 3135)<br>◯ Yverdon (Code SPSU 4106)<br>◯ Zollikerberg (Code SPSU 3131)<br>◯ Universitäts-Kinderklinik, Zürich (Code SPSU 3132)<br>◯ Spital Triemli, Zürich (Code SPSU 3133)
Reporting physician	__________________________________<br>(Please write your name in the box)
Age for children ≥ 1 month	__________________________________<br>(Please specify years and months, e.g. 2 years and 11 months = 2/11)
Age for children < 1 month	__________________________________<br>(Please specify the age in days)
Gestational age at birth if child is less than 1 year of age	__________________________________<br>(Please specify weeks and days, e.g. 38/5)
Birth weight in gram	__________________________________
Sex	◯ Male<br>◯ Female

Current weight in kg	______________________<br>(e.g. 14.5)
Current height in cm	______________________<br>(e.g. 123, if you don't know the height you can leave the field empty)
Country of birth	◯ Switzerland<br>◯ Germany<br>◯ Italy<br>◯ France<br>◯ Other<br>◯ Unknown
Which other country	______________________
Ethnicity	◯ Caucasian<br>◯ Black<br>◯ Asian<br>◯ Arabic<br>◯ Hispanic<br>◯ Other
Which other ethnicity	______________________
Migrant/asylum-seeker	◯ Yes<br>◯ No
Specify country of origin	◯ Eritrea<br>◯ Syria<br>◯ Afghanistan<br>◯ Turkey<br>◯ Georgia<br>◯ Other
Which other country of origin	______________________
Pre-existing medical conditions	☐ None<br>☐ Respiratory disease<br>☐ Immunodeficiency<br>☐ Cardiovascular disease<br>☐ Haematological/oncological disease<br>☐ Diabetes<br>☐ Other<br>(You can choose several answers if needed)
Which respiratory disease	______________________
Which immunodeficiency	______________________

Which cardiovascular disease	__________________________________
Which type of diabetes	__________________________________
Which other disease	__________________________________
Which haematological/oncological disease	__________________________________
Date of diagnosis of haematological/oncological disease	__________________________________
Specify treatment protocol of patient at time of SARS-CoV-2 diagnosis (e.g. AIEOP/BFM 2017 ect.)	__________________________________
Specify current treatment phase (e.g. induction or consolidation)	__________________________________
Stem cell transplant	◯ No/not applicable<br>◯ Yes, autologous<br>◯ Yes, allogenic
Date of stem cell transplant	__________________________________
Immunosuppressive drugs (at SARS-CoV-2 diagnosis)	☐ No<br>☐ Corticosteroids<br>☐ Biologicals<br>☐ Chemotherapy<br>☐ Other (e.g. cyclosporin, tacrolimus, mycophenolate, ect.)<br>(You can choose several answers if needed)
Name of corticosteroid	__________________________________
Dose of corticosteroid in mg	__________________________________
Time interval corticosteroids given	__________________________________<br>(Please specify times a day or week, ect.)
Specify name, dose and interval of each biological	____
Specify each drug of the chemotherapy with name, dose and interval	____

Specify each drug with name, dose and interval	__________________
MMR immunisation	◯ No<br>◯ One dose<br>◯ Two doses<br>◯ Unknown
Date of last MMR immunisation	__________________
BCG immunisation	◯ No<br>◯ Yes<br>◯ Unknown
Date of BCG immunisation	__________________
Influenza immunisation	◯ No<br>◯ One season<br>◯ Several seasons<br>◯ Unknown
Date of last influenza immunisation	__________________

Hospital admission

Admission to hospital	◯ Yes<br>◯ No
Date of hospital admission	__________________
Date of consultation	__________________
Reason for admission	◯ Suspected SARS-CoV-2 infection<br>◯ Confirmed SARS-CoV-2 infection<br>◯ Other reason
Other reason for admission	__________________
Previous admission for SARS-CoV-2 infection	◯ Yes<br>◯ No
Date of previous admission	__________________
Admitted from	◯ Home<br>◯ Other hospital<br>◯ Other

Admitted from which other place	__________________
Admission to intensive care unit	◯ Yes<br>◯ No
Date of intensive care unit admission	__________________
Reason for intensive care unit admission	◯ Respiratory failure<br>◯ Cardiac failure<br>◯ Other (please specify)
Other reason for intensive care unit admission	__________________
Date of intensive care unit discharge	__________________
Date of hospital discharge	__________________
Discharged to	◯ Home<br>◯ Other hospital<br>◯ Other<br>◯ Died
Discharged to which other hospital	__________________
Discharged to which other place	__________________

Symptoms

Respiratory distress/tachypnoea	◯ Yes ◯ No ◯ Not applicable
Oxygen saturation < 92%	◯ Yes ◯ No ◯ Not applicable
Date of onset of saturation < 92%	__________________
Fever (> 38°C)	◯ Yes ◯ No ◯ Not applicable
Date of onset of fever	__________________
Cough	◯ Yes ◯ No ◯ Not applicable
Pharyngitis/sore throat	◯ Yes ◯ No ◯ Not applicable
Rhinorrhea/nasal congestion	◯ Yes ◯ No ◯ Not applicable

Abdominal pain	◯ Yes ◯ No ◯ Not applicable
Vomiting	◯ Yes ◯ No ◯ Not applicable
Diarrhea	◯ Yes ◯ No ◯ Not applicable
Anosmia/dysgeusia	◯ Yes ◯ No ◯ Not applicable
Rash	◯ Yes ◯ No ◯ Not applicable
Specify rash (type, location)	__________________________________
Other	◯ Yes ◯ No ◯ Not applicable
Which other symptoms	__________________________________

Diagnostics

1st nasopharyngeal swab RT-PCR	◯ Not done ◯ Positive<br>◯ Negative
Date of 1st nasopharyngeal swab RT-PCR	____________________________
2nd nasopharyngeal swab RT-PCR	◯ Not done ◯ Positive<br>◯ Negative
Date of 2nd nasopharyngeal swab RT-PCR	____________________________
3rd nasopharyngeal swab RT-PCR	◯ Not done ◯ Positive<br>◯ Negative
Date of 3rd nasopharyngeal swab RT-PCR	____________________________
Detection of other viruses in nasopharyngeal swab	☐ Not done<br>☐ None found<br>☐ Influenza<br>☐ RSV<br>☐ Other<br>(You can choose several answers if needed)
Which other virus was detected in nasopharyngeal swab	____________________________
Throat swab RT-PCR	◯ Not done ◯ Positive<br>◯ Negative
Date of throat swab RT-PCR	____________________________

Tracheal aspiration RT-PCR	◯ Not done ◯ Positive ◯ Negative
Date of tracheal aspiration RT-PCR	__________________
BAL (bronchoalveolar lavage) RT-PCR	◯ Not done ◯ Positive ◯ Negative
Date of BAL (bronchoalveolar lavage) RT-PCR	__________________
Stool RT-PCR	◯ Not done ◯ Positive ◯ Negative
Date of stool RT-PCR	__________________
1st serology	◯ Not done ◯ Positive ◯ Negative
Date of 1st serology	__________________
Result of 1st serology: IgM with units	__________________
Result of 1st serology: IgA with units	__________________
Result of 1st serology: IgG with units	__________________
2nd serology	◯ Not done ◯ Positive ◯ Negative
Date of 2nd serology	__________________
Result of 2nd serology: IgM with units	__________________
Result of 2nd serology: IgA with units	__________________

Result of 2nd serology: IgG with units	____________________
1st chest x-ray	◯ Not done<br>◯ Normal<br>◯ Unilateral changes<br>◯ Bilateral changes<br>◯ ARDS<br>◯ Other
Which other findings were found on the 1st chest x-ray	____________________
Date of 1st chest x-ray	____________________
2nd chest x-ray	◯ Not done<br>◯ Normal<br>◯ Unilateral changes<br>◯ Bilateral changes<br>◯ ARDS<br>◯ Other
Which other findings were found on the 2nd chest x-ray	____________________
Date of 2nd chest x-ray	____________________
3rd chest x-ray	◯ Not done<br>◯ Normal<br>◯ Unilateral changes<br>◯ Bilateral changes<br>◯ ARDS<br>◯ Other
Which other findings were found on the 3rd chest x-ray	____________________
Date of 3rd chest x-ray	____________________
Echocardiography	◯ Not done ◯ Normal<br>◯ Abnormal
Which abnormal findings were found on echocardiography	____________________
Date of echocardiography	____________________
Was there another diagnostic done (e.g. CT)	◯ Yes ◯ No
Which other diagnostic was done, on what date and what was found	________________________

Treatment

Oxygen support	◯ Yes ◯ No
How many days on oxygen	__________________________________
High-flow oxygen	◯ Yes ◯ No
How many days on high-flow oxygen	__________________________________
CPAP or other NIV (NIV=non-invasive ventilation)	◯ Yes ◯ No
How many days on CPAP or other NIV	__________________________________
Mechanical ventilation	◯ Yes ◯ No
How many days on mechanical ventilation	__________________________________
Maximum FiO2 in %	__________________________________
Maximum flow rate in L/min	__________________________________
ECMO	◯ Yes ◯ No
How many days on ECMO	__________________________________
Inotropic support	◯ Yes ◯ No
How many days on inotropic support	__________________________________
Anti-inflammatory treatment	☐ No<br>☐ Corticosteroids<br>☐ Azithromycin<br>☐ Biologics (e.g. anakinra, tocilizumab, siltuximab, ect.)<br>☐ Immunoglobulins<br>☐ Other<br>(You can choose several answers if needed)
Specify dose, interval and dates corticosteroids was given	__________________________________
Specify dose, interval and dates azithromycin was given	__________________________________
Specify drug, dose, interval and dates of biological given	__________________________________

Specify drug, dose, interval and dates of immunoglobulins given	______________________
Which other anti-inflammatory drug was given and in which dose, interval and dates	______________________
Antiviral treatment	☐ No<br>☐ Hydroxychloroquine<br>☐ Lopinavir/ritonavir<br>☐ Remdesivir<br>☐ Other<br>(You can choose several answers if needed)
Specify dose, interval and dates hydroxychloroquin was given	______________________
Specify dose, interval and dates lopinavir/ritonavir was given	______________________
Specify dose, interval and dates remdesivir was given	______________________
Which other antiviral drug was given and in which dose, interval and dates	______________________

Complications

Complications	☐ No<br>☐ Vascular/Cardiac<br>☐ Neurologic<br>☐ Superinfection<br>☐ Other<br>(You can choose several answers if needed)
Which other complication	______________________
Specify vascular/cardiac complication	______________________
Specify neurological complication	______________________
Specify superinfection	______________________

Transmission / exposure

Exposure to presumed index case	◯ No<br>◯ Yes, SARS-CoV-2 confirmed<br>◯ Yes, without confirmation<br>(Please write down the person by whom the child was likely infected)
Specify relation and age (e.g. brother, 15 years)	______________________
Further cases in household	◯ No<br>◯ Yes, SARS-CoV-2 confirmed<br>◯ Yes, without confirmation<br>(Please do not include the index case if mentioned in the question before)
Specify relation and age (e.g. brother, 15 years)	______________________
Further cases outside household	◯ No<br>◯ Yes, SARS-CoV-2 confirmed<br>◯ Yes, without confirmation
Specify relation and age (e.g. brother, 15 years)	______________________
